# Integrated genomic and transcriptomic analysis of maize SRC2-like genes highlights divergent roles in abiotic stress responses

**DOI:** 10.3389/fpls.2026.1779434

**Published:** 2026-03-02

**Authors:** Huafeng Liu, Qianjin Zhang, Zihan Lu, Xiaomin Lu, Xiaomeng Shen, Yanyu Tian, Feiyu Ye, Chenchen Ma, Yazhou Deng, Xiang Guo, Xin Zhang, Liru Cao

**Affiliations:** The Shennong Laboratory, Grain Crops Research Institute, Henan Academy of Agricultural Sciences, Zhengzhou, Henan, China

**Keywords:** abiotic stress, C2 domains, maize, transcriptional regulation, ZmSRC2L

## Abstract

SRC2 (soybean genes regulated by cold 2) is a protein that contains a C2 domain and plays a vital role in plant stress responses. In this study, we identified a total of 15 *SRC2-like* genes (*ZmSRC2L1*–*ZmSRC2L15*) in maize and systematically characterized their molecular properties, genomic distribution, promoter features, subcellular localization, and expression under abiotic stress conditions. We confirmed that the *ZmSRC2L* genes are unevenly distributed across chromosomes 1–8, encoding proteins of 212–402 amino acids, with molecular weights (MWs) ranging from 21.93 to 42.52 kDa. These proteins retain a conserved C2_SRC2_like domain while exhibiting subfamily-specific variations. Promoter analysis revealed the presence of cis-elements enriched in light, hormones, and stress responses, and predicted regulation by multiple transcription factor families, particularly ERF and MYB. Subcellular localization analysis revealed that the ZmSRC2L proteins are distributed across multiple cellular compartments. Transcriptome and RT-qPCR analyses demonstrate that gene expression is tissue-specific under stresses such as drought, heat, and cold. Functional validation using *Ds* insertion mutants indicated that the loss of *ZmSRC2L2* specifically impaired drought tolerance without affecting responses to heat or cold. Overall, these results provide a comprehensive framework for understanding the role of the *ZmSRC2L* family in abiotic stress responses and highlight *ZmSRC2L2* and other members as promising targets for enhancing maize stress resistance.

## 1 Introduction

Maize is a key crop for food, forage, and the production of industrial compounds (Godfray et al., 2010; Andorf et al., 2019). However, adverse environmental factors, including extreme temperatures (both heat and cold), water scarcity, and salt stress, have been shown to detrimentally affect maize growth across various developmental stages, leading to significant reductions in both yield quantity and quality (Bashir et al., 2019). With the continuous growth of the global population (United Nations, 2023), increasing pressure is being placed on agricultural systems, making the impacts of environmental change on crop productivity an increasingly critical global concern.

Plants employ a diverse array of signaling processes in response to abiotic stresses, many of which involve the perception of signals at the plasma membrane followed by their transduction to the cytoplasm and nucleus. A key aspect of membrane-associated signaling is mediated by peripheral membrane proteins containing C2 domains. The C2 domain was first identified as one of four conserved modules (C1–C4) within the classical Ca²^+^-dependent isoforms (α, β, and γ) of mammalian protein kinase C (PKC) (Coussens et al., 1986; Knopf et al., 1986; Nishizuka, 1988). The C2 domain is approximately 130 amino acids in length and is capable of binding phospholipids in a Ca²^+^-dependent manner (Kaibuchi et al., 1989; Nalefski and Falke, 1996; Wang, 2002). In rice, the small protein OsERG1 contains a single C2 domain and is induced by treatment with a fungal elicitor, which results in the protein binding to phospholipid vesicles in a Ca²^+^-dependent manner (Kim et al., 2003). Similarly, in mung beans (*Vigna radiata L.*), the C2 domain of V3-PLC3, a putative plasma membrane-localized phosphoinositide-specific phospholipase C, plays a crucial role in the translocation of the protein to the membrane in response to abiotic stress (Kim et al., 2004; Wang, 2002).

SRC2 (soybean genes regulated by cold 2) is a C2 domain-containing protein in soybean, initially identified as being regulated by cold stress (Takahashi and Shimosaka, 1997). Its homolog in pepper, CaSRC2-1, is distinguished by specific conserved amino acids within the C2 domain. The transcription of *CaSRC2–1* is upregulated under biotic stress conditions (Kim et al., 2008). Further studies have revealed that pepper *SRC2–1* is essential for elicitor Phytophthora capsici INF1 (PcINF1)-induced immunity in pepper plants, functioning as an interacting partner of *PcINF1* (Liu et al., 2015). Recent research has also shown that the *SRC2* gene plays a role in Cucumber mosaic virus (CMV) infection, where silencing of *SRC2* in Nicotiana benthamiana increased susceptibility to CMV, suggesting that *SRC2* may be involved in resistance to the virus (Saikia et al., 2025). Moreover, tobacco NbSRC2 interacts with the 17-kDa protein (17K) of Barley yellow dwarf viruses (BYDVs) to influence viral infection in tobacco by regulating reactive oxygen species (ROS) production (Chen et al., 2021). Further investigations revealed that barley SRC2 (HvSRC2) interacts with the BYDV-GAV 17K protein both *in vivo* and *in vitro*, with 17K promoting the accumulation of HvSRC2 to regulate plant defense pathways, facilitating BYDV infection (Wang et al., 2025). *AtSRC2* is a novel activator of Ca²^+^-dependent *AtRbohF*-mediated ROS production and may play a role in cold responses (Kawarazaki et al., 2013). However, the functional exploration of the *SRC2* gene in abiotic stress remains relatively limited.

However, most previous studies focused on individual *SRC2* genes or stress-induced expression rather than systematic evolutionary and functional comparisons across Poaceae, leaving the diversification of *SRC2-like* (*SRC2L*) genes in cereals largely unexplored. In particular, the roles of *SRC2L* genes in maize, a key cereal crop with a highly complex genome, have not yet been clearly defined. To address this knowledge gap, we conducted a genome-wide identification of 15 maize *SRC2L* genes (*ZmSRC2L1*–*ZmSRC2L15*) and analyzed their structures, conserved domains, and phylogenetic relationships to clarify their evolution within Poaceae. We further examined their subcellular localization, tissue-specific expression, and responses to abiotic stress. In addition, a *Ds* insertion mutant of *ZmSRC2L2* (*zmsrc2l2^Ds^*) was characterized to verify its role in stress tolerance. Collectively, this study provides the first comprehensive genome-wide overview of *SRC2-like* genes in maize, elucidates their evolutionary divergence within Poaceae, and offers novel insights into the molecular basis of abiotic stress adaptation in cereals.

## 2 Materials and methods

### 2.1 Plant materials, growth conditions and stress treatments

The plant materials used in this study included the maize inbred line B73 and the *Ac/Ds* insertion mutant. Both lines were grown in growth chambers under controlled conditions with a 14-hour light/10-hour dark photoperiod, day/night temperatures of 26 °C/22 °C, and 40% relative humidity until the three-leaf stage. For the drought treatment of B73, watering was continued for the control group but withheld for the drought-stress group. The intensity of drought stress was quantitatively defined based on soil water content (SWC). “Mild drought” was imposed when SWC decreased to approximately 35–40% of field capacity, whereas “severe drought” corresponded to SWC ≤ 20% of field capacity, as described by Kwasniewski et al. (2016). Leaf and root tissues were sampled from both groups at two critical time points: 3 days (mild) and 7 days (severe) after the initiation of drought stress. For the temperature treatments, seedlings were exposed to 40 °C (heat) or 4 °C (cold) for 1, 3, 6, 12, and 24 hours. These temperatures were selected according to previously published studies on maize stress physiology, representing physiologically relevant conditions that induce stress responses without causing irreversible tissue damage (Bashir et al., 2019). Control plants were maintained at 26 °C/22 °C (day/night). Tissue samplings were performed at 1-, 3-, 6-, 12-, and 24-hours post-treatment. All treatments included three biological replicates, each consisting of a pool of three individual plants. The *zmsrc2l2^Ds^* mutant was subjected to drought, heat and cold stress for 3 days at the three-leaf stage, respectively. The leaf relative water content (RWC) of plants grown under normal and abiotic stresses were measured in accordance with Kwasniewski’s method (Kwasniewski et al., 2016). The activities of the antioxidant enzymes superoxide dismutase (SOD), peroxidase (POD), and catalase (CAT) were measured using commercial analytical kits (Nanjing Jiancheng Bioengineering Institute). Meanwhile, the content of chlorophyll (Chl) was determined according to Nita et al. (2015). Free proline (Pro) was extracted from approximately 100 mg of leaf tissue with 3 mL of 3% sulfosalicylic acid and quantified using 2.5% acidified ninhydrin, as described by Teklić et al. (2010). To ensure reliability, two independent drought experiments were conducted.

### 2.2 Identification of the *SRC2-like* genes family

The whole genome sequences and annotation files for *Zea mays* (Taxid: 4577), *Oryza sativa* (4530), *Sorghum bicolor* (4558), *Setaria italica* (4555), *Setaria viridis* (4556), and *Arabidopsis thaliana* (3702) were downloaded from Phytozome (V13, https://phytozome-next.jgi.doe.gov/), while the AtSRC2 protein sequences were obtained from TAIR (https://www.arabidopsis.org). All gene protein sequences of the six species were consolidated into a single file to construct a BLASTP database. The *SRC2L* genes in these species were identified through two rounds of BLASTP. In the first round, the AtSRC2 protein sequence was used to search for potential SRC2Ls using Auto BlastTwo Sequences Set (E-value=1e-5). In the second round, the potential SRC2L proteins sequences from the first round were further identified using BLASTP against the constructed database. Candidate *SRC2L* genes were then submitted to NCBI-CDD (https://www.ncbi.nlm.nih.gov/Structure/bwrpsb/bwrpsb.cgi) and Pfam (http://pfam.xfam.org/search) for confirmation of the presence of the C2_SRC2_like (cd04051) conserved domain. For *ZmSRC2L* genes, ExPASy-ProtParam (https://web.expasy.org/protparam/) was used to predict the coding sequence (CDS) length, isoelectric point (pI), and molecular weight (MW).

### 2.3 *ZmSRC2Ls* multiple sequence alignment and chromosome location analysis

Multiple sequence alignment of *ZmSRC2L* genes was calculated by Jalview (Clamp et al., 2004) and TBtools (Chen et al., 2020) was used to confirm the chromosome location and draw the chromosome distribution map of all *ZmSRC2L* genes family.

### 2.4 *ZmSRC2Ls* gene structure and conserved domains analysis

The analysis and visualization of gene structure and conserved domain of the *ZmSRC2Ls* were realized through Batch-Search (https://www.ncbi.nlm.nih.gov/Structure/bwrpsb/bwrpsb.cg) and TBtools (Chen et al., 2020). To further understand the *ZmSRC2Ls* function, the protein sequences were submitted to MEME program to analyze the conserved motifs. And the parameters were as follows: the number of repetitions was set to zero or one, and the maximum number of motifs was set to 3 (Bailey et al., 2009).

### 2.5 Phylogenetic analysis

The correctly validated SRC2 family protein sequences of *Zea mays*, *Oryza sativa*, *Sorghum bicolor*, *Setaria italica*, *Setaria viridis*, and *Arabidopsis thaliana* were obtained using the samtools faidx command on the annotated protein sequence files. A phylogenetic tree of the protein sequences from ZmSRC2Ls, OsSRC2Ls, SbSRC2Ls, SiSRC2Ls, and SvSRC2Ls was constructed using the maximum likelihood method with 1000 bootstrap replicates in MEGA7.0.

### 2.6 Estimation of Ka, Ks and Ka/Ks values

Orthologous gene pairs between *Zea mays* and *Setaria italica*, *Sorghum bicolor*, *Setaria viridis* and *Oryza sativa* were identified using reciprocal best BLAST hits (RBH) with a cutoff E-value of 1e-5. Coding sequences (CDS) of each orthologous pair were aligned using MAFFT v7 (Katoh and Standley, 2013) with codon-level refinement. The aligned codon matrices were then subjected to evolutionary rate estimation using the Yang-Nielsen (YN00; Yang and Nielsen, 2000) model implemented in PAML v4.9 (Yang, 2007). For each gene pair, nonsynonymous substitution rate (Ka), synonymous substitution rate (Ks), and their ratio (Ka/Ks) were calculated. Gene pairs with unreliable Ks estimates (Ks > 5 or Ks = 0) were removed to avoid saturation artifacts. Boxplots of Ka, Ks and Ka/Ks distributions were generated using R v4.3 with ggplot2.

### 2.7 Promoter cis-acting element and transcription factor prediction

For *ZmSRC2Ls*, the 2000 bp region upstream of the CDS was extracted as a promoter using the seqkit package (Shen et al., 2024). Promoter cis-acting elements were predicted using PlantCare (Lescot et al., 2002) and visualized with TBtools after statistical screening. Transcription factors (TFs) were then predicted using PlantTFDB (Jin et al., 2014, 2017) and displayed using Cytoscape (Shannon et al., 2003).

### 2.8 *ZmSRC2Ls* gene family cloning and plasmid construction

Cloning primers for all *ZmSRC2Ls* CDS were designed using Primer Premier 6. cDNA from B73 leaves was used as a template to amplify the *ZmSRC2Ls* CDS. The 50 µL PCR system included: 25 µL of 2× Phanta^®^Max Buffer, 1 µL of Phanta^®^ Max Super-Fidelity DNA Polymerase, 1 µL of dNTP, 2 µL of cDNA, 2 µL of each primer (10 µM), 17 µL of ddH_2_O. The PCR program was as follows: pre-denaturation at 95 °C for 3 min, denaturation at 94 °C for 15 s, annealing at 55 °C for 15 s, extension at 72 °C for 1 min, for 35 cycles, followed by a final extension at 72 °C for 5 min. The PCR products were purified using the Gel Extraction Kit (OMEGA), then ligated into the pEasy-Blunt3 vector (TransGen Biotech) and sequenced. For subcellular localization analysis, the CDS of *ZmSRC2Ls* without the stop codon was amplified and ligated into the transient expression pCAMBIA1300 vector with a GFP fluorescent label and a CaMV 35S promoter. All amplification primers for plasmid construction are listed in **Supplementary Table S1**.

### 2.9 Subcellular localization analysis

For the maize protoplast subcellular localization experiment, B73 maize seeds were germinated at a constant temperature (25 °C) in continuous darkness for 1–2 weeks until yellowed seedlings were obtained. The leaves of the yellowed seedlings were harvested, cut into approximately 1 mm wide strips, and placed in a protoplast extraction buffer containing 0.6 M mannitol, 10 mM KCl, 5 mM CaCl_2_, 1% Pectolyase Y-23, and 0.5% Cellulase Onozuka R-10. The mixture was gently shaken at 25 °C for 1–2 hours to degrade the cell walls and release the protoplasts. After lysis, the mixture was filtered through a 150 μm cell strainer to remove undissolved clumps, followed by centrifugation at 800 g for 5 minutes and washing with 0.6 M mannitol solution. This washing step was repeated 2–3 times to remove impurities. Subsequently, protoplasts were transformed using PEG-mediated transformation by mixing with either empty pCAMBIA1300-GFP or pCAMBIA1300-ZmSRC2Ls-GFP plasmid vectors (10-50 μg/mL), and incubated with 2.5% PEG 4000 solution on ice for 20–30 minutes. After transformation, the protoplasts were transferred to a recovery medium containing 0.6 M mannitol, centrifuged, and incubated in the dark at 25 °C for 24 hours to allow recovery. Finally, transformed protoplasts were analyzed using confocal or fluorescence microscopy to observe the fluorescence signals of the reporter gene, enabling the analysis of the subcellular localization of the target protein.

### 2.10 RNA sequencing and RT‐qPCR analysis

The total RNA from all samples was extracted using the HiPure HP PlantRNA Kit (Magen). The RNA integrity, concentration, and quality were assessed using 1% agarose gel electrophoresis and a NanoDrop ND1000 (Thermo Scientific). cDNA libraries were constructed and high-throughput sequencing was performed by Biomarker Technologies Corporation. A total of 1.95 billion paired-end reads were generated from each of the 84 cDNA libraries (**Supplementary Table S2**). The resulting high-quality reads were aligned and mapped to the maize B73_V4 genome using HISAT2 (Kim et al., 2019), with over 85.42% of the reads uniquely mapped to the B73_V4 maize reference genome (**Supplementary Table S2**). Read counting was performed using the edgeR package (Robinson et al., 2010). Differentially expressed genes (DEGs) were identified using the DESeq2 package (Love et al., 2014). Gene expression levels were quantified based on fragments per kilobase of transcript per million fragments mapped (FPKM). For RT-qPCR analysis, 0.5 µg of total RNA was used to synthesize cDNA following the manufacturer’s instructions for the HiScript II Reverse Transcript Kit (Vazyme). RT-qPCR primers for all *ZmSRC2Ls* were designed across introns using NCBI Primer-BLAST (https://www.ncbi.nlm.nih.gov/tools/primer-blast/), based on the gene structure. The 10 µL RT-qPCR reaction system, according to the ChamQ Universal SYBR qPCR Master Mix (Vazyme) instructions, included 2 µL of template cDNA, 0.4 µL of each primer (10 µM), 7.2 µL of ddH_2_O. The reaction program was as follows: pre-denaturation at 95 °C for 30 s; denaturation at 95 °C for 15 s, annealing at 60 °C for 20 s, extension at 72 °C for 10 s, for 40 cycles; followed by melting curve analysis from 65–95 °C. Three biological replicates were performed for each sample, with three technical repetitions per biological replicate. *UBQ* was used as the reference gene under all conditions. Relative expression levels of all *ZmSRC2Ls* under different conditions were calculated using the 2^-ΔΔCt^ method (Livak and Schmittgen, 2001).

## 3 Results

### 3.1 Identification and characterization of C2 domain-containing *SRC2-Like* genes in maize

To identify members of the *ZmSRC2L* gene family in maize, we first performed BLASTP searches against the maize proteome using the full-length amino acid sequence of the Arabidopsis gene *AtSRC2* as the query. Candidate proteins were subsequently examined for the presence of the conserved C2_SRC2_like domain (cd04051) using both the Pfam and NCBI Conserved Domain Database (CDD), resulting in the identification of 15 *ZmSRC2L* genes, which were named *ZmSRC2L1*–*ZmSRC2L15* based on their physical positions on the maize chromosomes. The coding sequence (CDS) lengths of the *ZmSRC2L* genes range from 312 to 1,170 bp, encoding proteins of 212–402 amino acids. The predicted molecular masses of *ZmSRC2L1*, *ZmSRC2L7*, *ZmSRC2L8*, and *ZmSRC2L11* do not exceed 30 kDa, whereas *ZmSRC2L9* has the greatest molecular mass (41.52 kDa). The theoretical isoelectric points (pI) of the 15 ZmSRC2L proteins vary from 4.67 to 10.04. All proteins exhibit negative GRAVY values, indicating that they are hydrophilic. The detailed physicochemical properties of these proteins are summarized in **Table 1**.

**Table 1:** Identification and characterization of ZmSRC2L genes family in maize.

Gene name	Gene ID	CHr	Start	End	Strand	Number of amino acid	Molecular weight	Isoelectric point	Instability Index	Aliphatic index	GRAVY	Number of TMHs
ZmSRC2L1	Zm00001d031892	1	205,442,994	205,443,842	+	282	29416.58	4.67	42.89	69.65	-0.455	0
ZmSRC2L2	Zm00001d004483	2	114,559,829	114,560,905	–	358	38067.98	9.81	71.66	69.19	-0.395	0
ZmSRC2L3	Zm00001d005315	2	169,380,169	169,381,164	+	331	33574.78	5.02	38.47	62.63	-0.469	1
ZmSRC2L4	Zm00001d040838	3	69,660,612	69,661,469	+	285	30674.81	7.64	44.04	71.3	-0.221	0
ZmSRC2L5	Zm00001d042214	3	156,317,221	156,318,186	+	321	32859.75	8.46	45.43	64.39	-0.375	1
ZmSRC2L6	Zm00001d043082	3	188,683,547	188,685,059	–	315	34294.49	7.76	38.31	63.11	-0.503	0
ZmSRC2L7	Zm00001d043990	3	215,697,796	215,698,632	–	278	29261.44	9.29	66.24	87.09	-0.123	0
ZmSRC2L8	Zm00001d051926	4	174,741,981	174,744,018	–	249	26559.25	10.04	53.34	86.99	-0.326	0
ZmSRC2L9	Zm00001d052662	4	196,294,664	196,295,872	–	402	41521.48	6.57	74.59	53.36	-0.431	0
ZmSRC2L10	Zm00001d016635	5	170,840,257	170,841,486	+	316	33839.23	6.05	63.08	73.23	-0.328	0
ZmSRC2L11	Zm00001d037892	6	141,260,676	141,261,314	–	212	21932.06	9.86	50.75	85.28	-0.065	0
ZmSRC2L12	Zm00001d019750	7	56,217,746	56,218,720	+	324	33213.55	5.12	33.07	65.46	-0.461	1
ZmSRC2L13	Zm00001d022478	7	178,396,449	178,397,393	–	314	32556.99	9.06	41.02	83.12	-0.083	0
ZmSRC2L14	Zm00001d011919	8	164,591,701	164,592,765	+	354	36149.38	8.68	51.27	60.62	-0.403	1
ZmSRC2L15	Zm00001d012336	8	172,697,736	172,698,849	–	327	35193.5	9.02	64.04	63.27	-0.711	0

**Figure 1:**
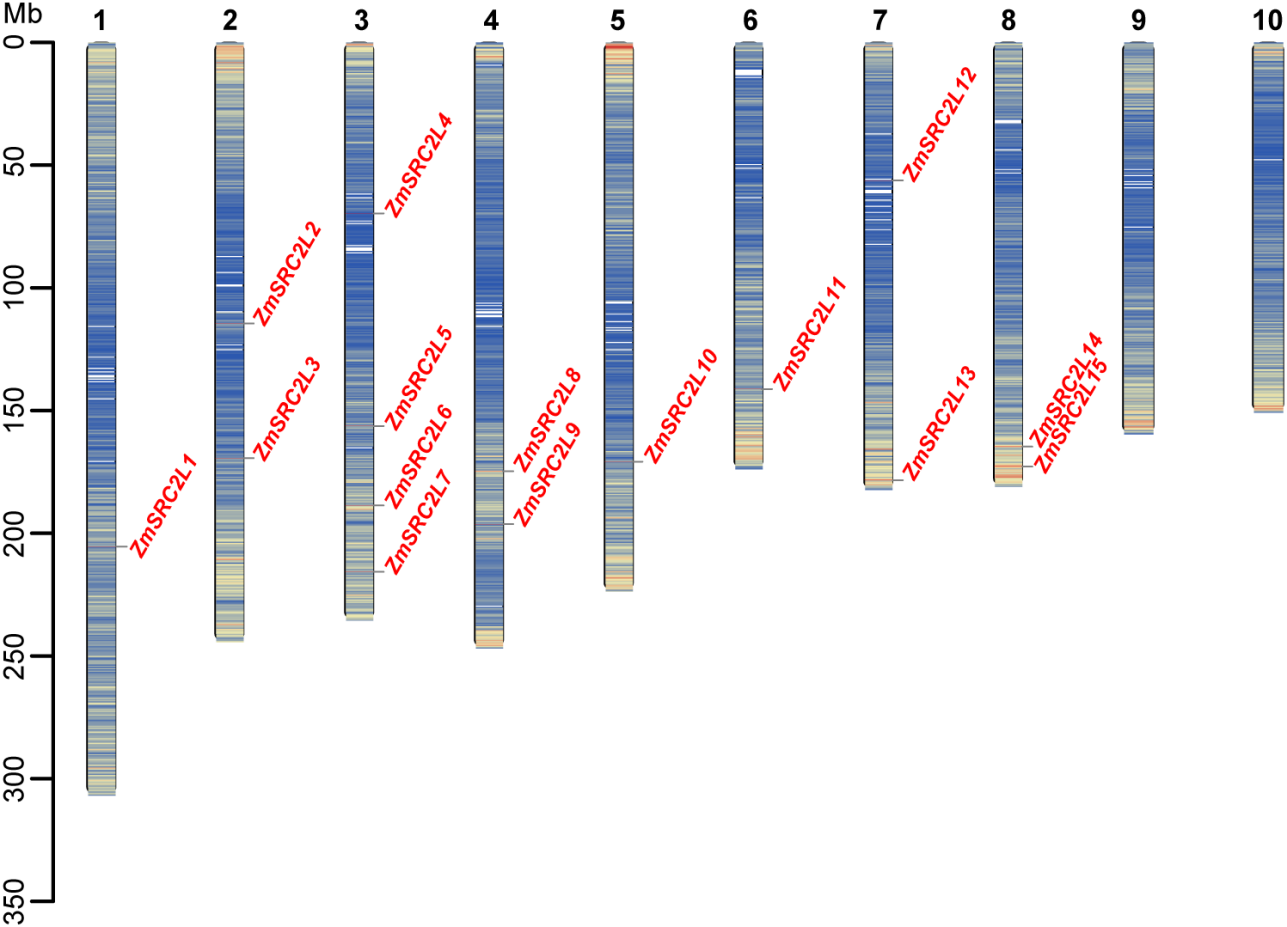
Chromosomal location of *ZmSRC2L* genes family in maize.

To characterize the chromosomal distribution pattern of *ZmSRC2L* genes in maize, we mapped their genomic loci using the reference genome sequence. The *ZmSRC2L* genes were unevenly distributed across chromosomes 1–8. A single *ZmSRC2L* gene was found on chromosomes 1, 5, and 6, whereas chromosomes 2, 4, 7, and 8 each harbored two *ZmSRC2L* genes. Chromosome 3 contained four *ZmSRC2L* genes. No *ZmSRC2L* genes were detected on chromosomes 9 and 10 (**Figure 1**).

**Figure 2:**
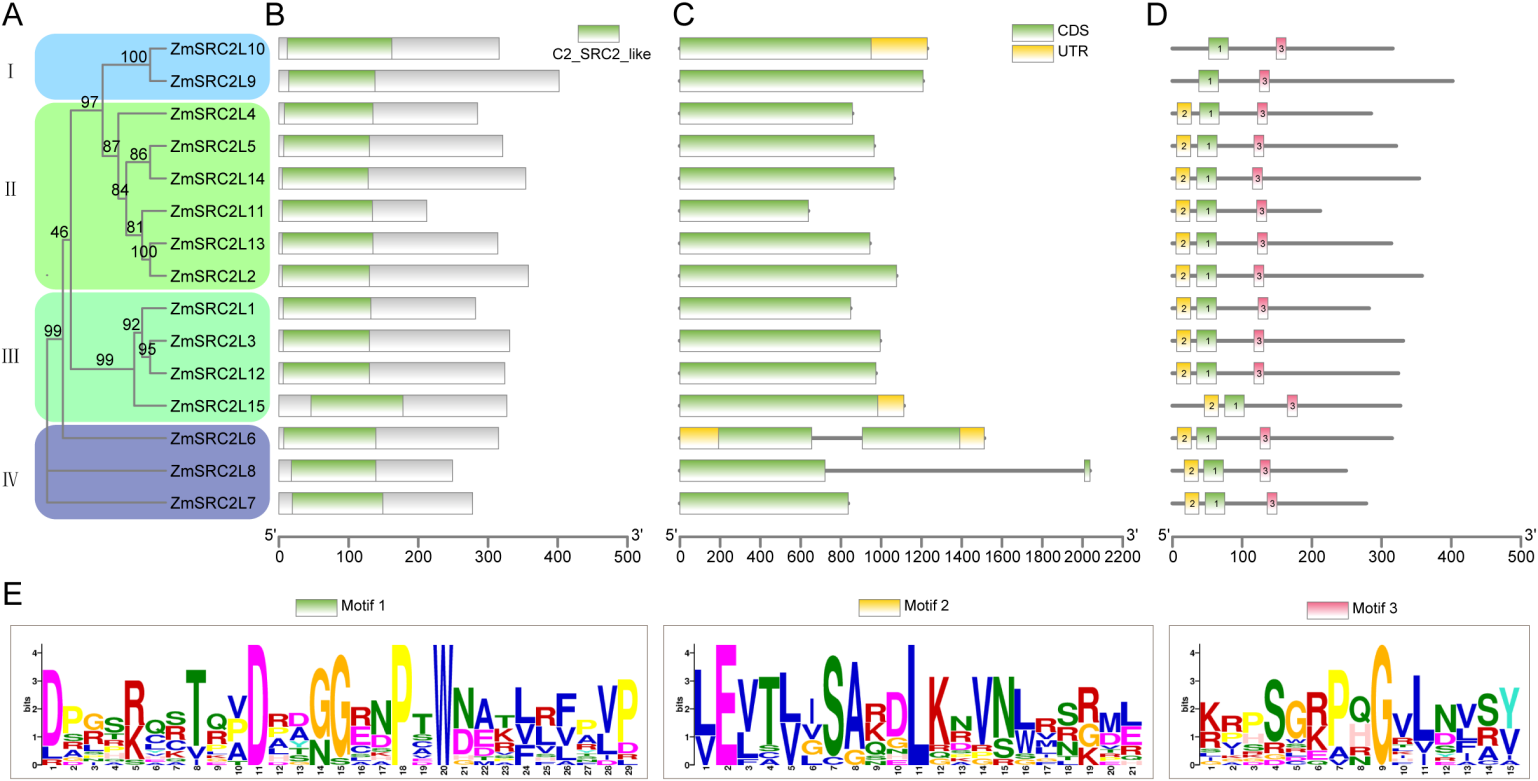
Phylogenetic relationship, gene structure, conserved domains and motifs of ZmSRC2Ls proteins in maize. **(A)** The phylogenetic tree of all ZmSRC2Ls proteins was constructed using the maximum likelihood method with 1000 bootstrap replicates. **(B)** Distribution of the conserved C2_SRC2_like domain in ZmSRC2L proteins, as identified using the NCBI Conserved Domain Database (CDD). **(C)** The UTR, CDS and intron organization of ZmSRC2Ls. The yellow boxes represent UTRs, green boxes represent CDSs and thin black lines represent introns. **(D)** The conserved motifs in ZmSRC2L proteins were identified using the MEME suite. Motifs 1–3 are denoted by distinct colored boxes, and their corresponding sequence logos are displayed in panel. **(E)** In the sequence logos, the y-axis (in bits) reflects the relative frequency of each amino acid.

A comprehensive analysis of the phylogenetic relationships, conserved domains, gene structures, and motif compositions of the *ZmSRC2L* genes revealed both conserved and divergent features within this family. The phylogenetic tree grouped the 15 *ZmSRC2L* genes into four subfamilies (I–IV) (**Figure 2A**). Conserved domain analysis showed that all ZmSRC2L proteins share a characteristic core domain that are likely critical for their biochemical and functional specificity (**Figure 2B**). Gene structure analysis indicated that, with the exception of *ZmSRC2L6* and *ZmSRC2L8*, which contain introns, the remaining 13 genes are intronless (**Figure 2C**), suggesting a relatively compact gene architecture for most family members. Motif analysis further identified three conserved motifs. Motifs 1 and 3 were present in all ZmSRC2L proteins, implying a central role in maintaining basic protein function, whereas motif 2 was absent from *ZmSRC2L9* and *ZmSRC2L10* (**Figures 2D, E**), which may reflect diversification driven by gene duplication and subsequent functional specialization in maize. Collectively, these results suggest that *ZmSRC2L* genes from different subfamilies retain a conserved functional core while exhibiting subfamily-specific divergence.

### 3.2 Phylogenetic, collinearity, and evolutionary analysis of SRC2L genes in Poaceae

**Figure 3:**
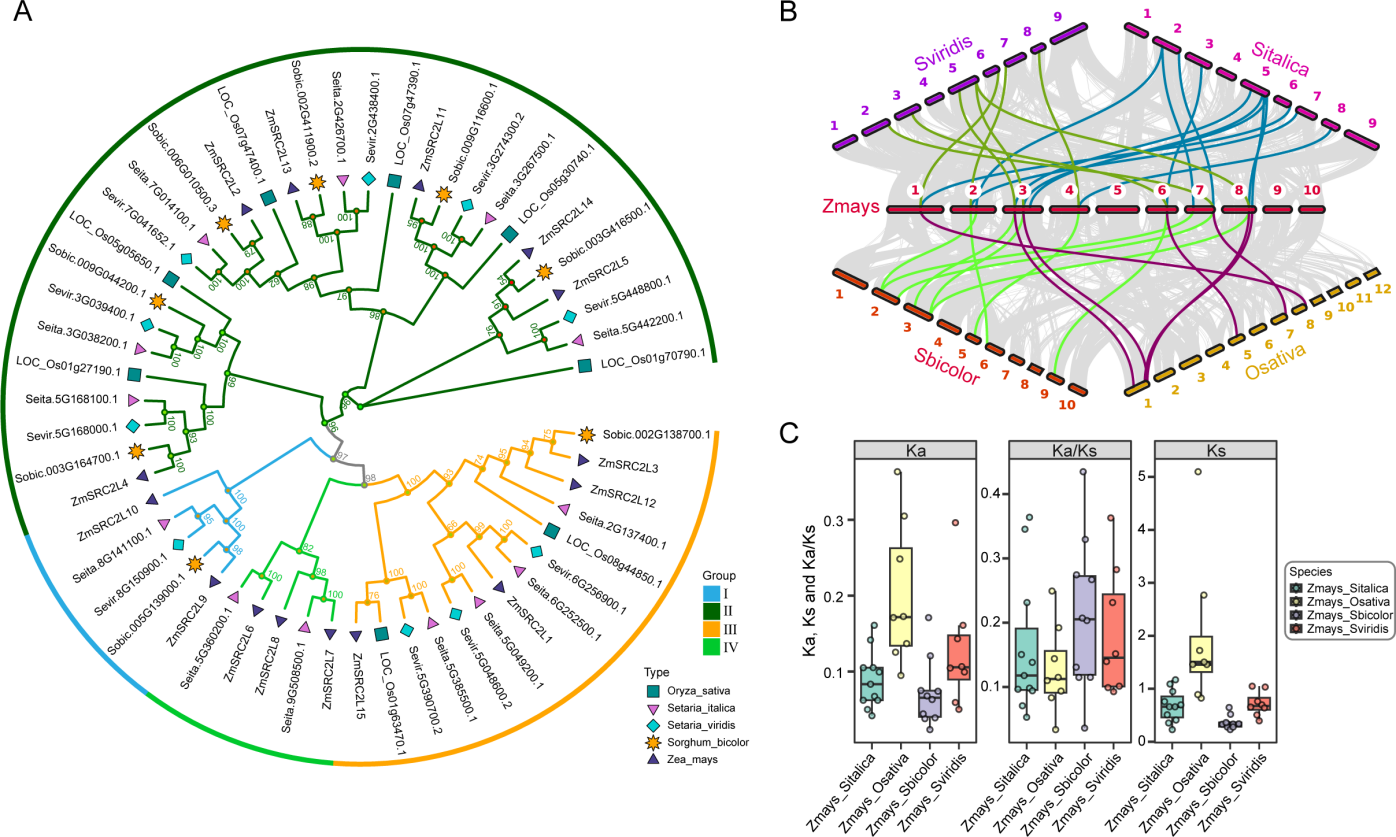
Phylogenetic relationships, collinearity, and evolutionary analysis of *SRC2L* genes in Poaceae. **(A)** Phylogenetic tree constructed from the SRC2L protein sequences of five Poaceae species (*Oryza sativa*, *Setaria italica*, *Setaria viridis*, *Sorghum bicolor*, and *Zea mays*). The *SRC2L* genes are grouped into four major group (I–IV). Different colors represent distinct species. **(B)** Collinearity analysis between *Z. mays SRC2L* genes and their orthologs in the other four Poaceae species. Colored lines represent syntenic gene pairs. **(C)** Comparative analysis of nonsynonymous (Ka), synonymous (Ks), and Ka/Ks ratios between *Z. mays SRC2L* genes and their orthologs in *O. sativa*, *S. italica*, *S. viridis*, and *S. bicolor*. The Ka/Ks ratios reveal the selective pressures acting on *SRC2L* genes during the evolution of Poaceae.

To investigate the evolutionary relationships of the *SRC2L* gene family in Poaceae, a phylogenetic tree was constructed using SRC2L protein sequences from representative species, including *Oryza sativa*, *Setaria italica*, *Setaria viridis*, *Sorghum bicolor*, and *Zea mays* (**Figure 3A****;****Supplementary Table S3**). The results revealed that all SRC2L proteins were clustered into four distinct groups (I–IV), suggesting an ancient duplication and divergence event prior to the diversification of these grass species. Notably, the *SRC2L* genes in maize were distributed across all four groups, indicating that multiple SRC2L copies have been retained throughout maize genome evolution. In contrast, *O. sativa* and *S. bicolor* each contained fewer *SRC2L* genes, implying partial gene loss or lineage-specific contraction.

To further explore the evolutionary conservation of these genes, a collinearity analysis was performed between *Z. mays* and the other four species (**Figure 3B**). Extensive syntenic relationships were identified, particularly between *Z. mays* and S*. italica* as well as *S. viridis*, reflecting a high degree of genomic conservation among these grasses. Several collinear blocks were also detected between *Z. mays* and *O. sativa*, although fewer orthologous pairs were observed, suggesting that genomic rearrangements and segmental duplication events might have occurred during species divergence.

To assess the selection pressures acting on the *SRC2L* gene family, the nonsynonymous (Ka), synonymous (Ks), and Ka/Ks ratios were calculated for each orthologous gene pair (**Figure 3C**). The majority of Ka/Ks ratios were less than 1.0, indicating that the *SRC2L* genes have mainly undergone purifying selection to maintain their conserved biological functions. However, a few orthologous pairs exhibited relatively higher Ka/Ks values, suggesting that positive selection may have driven functional diversification in certain *SRC2L* members, especially within the maize lineage. Collectively, these results demonstrate that the *SRC2L* gene family is evolutionarily conserved across Poaceae species but has also experienced species-specific expansion and divergence.

### 3.3 Promoter cis-element profiling and transcription factor prediction for *ZmSRC2L* genes

**Figure 4:**
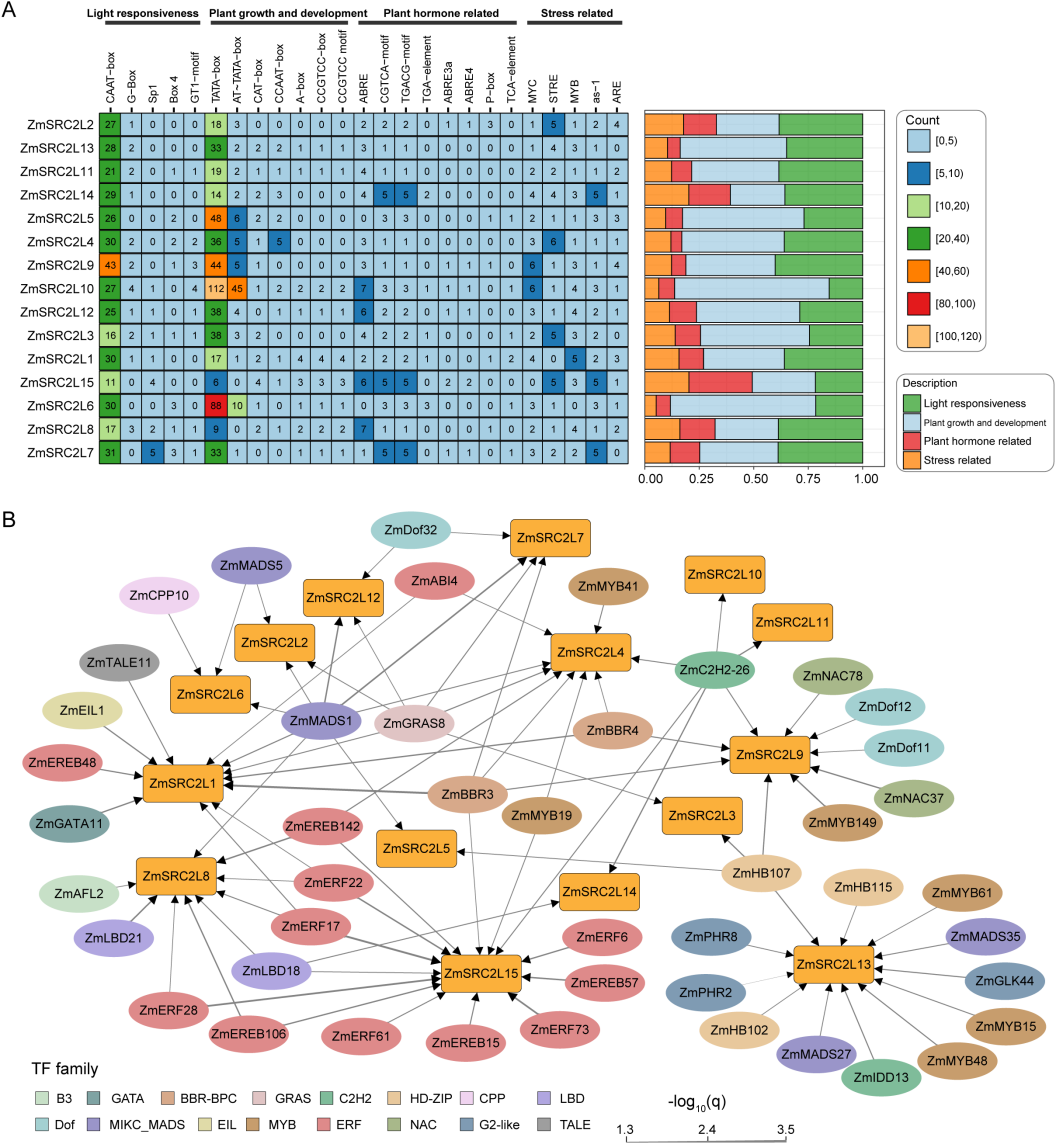
Prediction of cis-acting elements and regulatory network analysis of *ZmSRC2L* promoters. **(A)** Distribution and functional classification of predicted cis-acting elements in the 2-kb promoter regions of *ZmSRC2L* genes. **(B)** Regulatory network between *ZmSRC2L* genes and potential transcription factors. Different colors represent distinct TF families. The thickness of each connecting line indicates the confidence level (or probability) of regulatory interaction between TFs and *ZmSRC2L* genes.

Analysis of the cis-regulatory elements in the 2-kb promoter regions of the 15 *ZmSRC2L* genes revealed considerable variation in both the types and abundance of elements, reflecting functional diversity within the gene family (**Figure 4A****;****Supplementary Table S4**). Light-responsive elements, such as CAAT-box, G-box, were abundant in most promoters, with *ZmSRC2L7*, *ZmSRC2L8*, and *ZmSRC2L9* showing the highest proportions, suggesting a key role in light-mediated transcriptional regulation. Developmental cis-regulatory elements such as TATA-box, CAT-box, A-box, and CCAAT-box were present across all genes, with *ZmSRC2L6* and *ZmSRC2L10* exhibiting notably higher counts, indicating their potential involvement in stage-specific regulation. Hormone-responsive elements, including ABRE, CGTCA-motif, TGACG-motif, and P-box, were enriched in several promoters, particularly in *ZmSRC2L2*, *ZmSRC2L7*, *ZmSRC2L8*, *ZmSRC2L14* and *ZmSRC2L15*, suggesting that these genes are involved in hormone signaling pathways, such as ABA, JA, and GA responses (**Figure 4A****;****Supplementary Table S4**). In addition, stress-related elements, including MYB, MYC, and ARE, were also notably enriched in *ZmSRC2L2*, *ZmSRC2L8*, *ZmSRC2L14*, and *ZmSRC2L15*, suggesting their involvement in mediating responses to environmental stresses, potentially through plant hormone signaling pathways. These results suggest that *ZmSRC2L* genes play distinct roles in plant hormone and environmental stress responses.

The prediction of transcription factors (TFs) regulating the *ZmSRC2L* gene family aims to identify key TFs that potentially modulate the expression of these genes in response to environmental and developmental signals. To this end, potential TFs binding to the *ZmSRC2Ls* promoters were predicted using PlantTFDB (**Figure 4B****;****Supplementary Table S5**). A total of 16 TF families were predicted to potentially regulate the *ZmSRC2L* genes. Among them, 12 ERF TFs were predicted to regulate *ZmSRC2L1*, *ZmSRC2L4*, *ZmSRC2L8* and *ZmSRC2L15*, six MYB TFs were linked to the regulation of *ZmSRC2L4*, *ZmSRC2L9*, *ZmSRC2L13* and *ZmSRC2L15*, and four MADS TFs were associated with the regulation of the majority of *ZmSRC2L* genes (**Figure 4B****;****Supplementary Table S5**). Additionally, three Dof TFs were predicted to regulate *ZmSRC2L7*, *ZmSRC2L9*, *ZmSRC2L12*, and three G2-like TFs were found to regulate only *ZmSRC2L13*. Among the *ZmSRC2L* genes, *ZmSRC2L15* was predicted to be regulated by the most TFs, with a total of 14, 71.43% (10/14) of which were ERF TFs. Notably, *ZmERF17* showed the highest regulatory potential for *ZmSRC2L15* (*P* < 0.0001)(**Supplementary Table S5**), suggesting a significant role for *ZmSRC2L15* in maize developmental processes. Furthermore, ZmSRC2L13 is predicted to be regulated by 12 TFs from five families, including MYB, HD-ZIP, MADS, C2H2, and G2-like, suggesting that this gene may be involved in regulating plant developmental processes and responding to environmental changes through multiple signaling pathways. Taken together, these analyses provide a foundation for further research on the functions of *ZmSRC2L* genes in plant growth, development, and stress responses.

### 3.4 Subcellular location of ZmSRC2L proteins

To investigate the subcellular localization of the ZmSRC2L proteins, each *ZmSRC2L* coding sequence was fused in-frame to the N-terminus of GFP to generate the pGreen-ZmSRC2L fusion construct. The constructs, lacking stop codons, were subsequently inserted into the pCAMBIA1300 vector under the control of the CaMV 35S promoter for transient expression in maize protoplasts.

**Figure 5:**
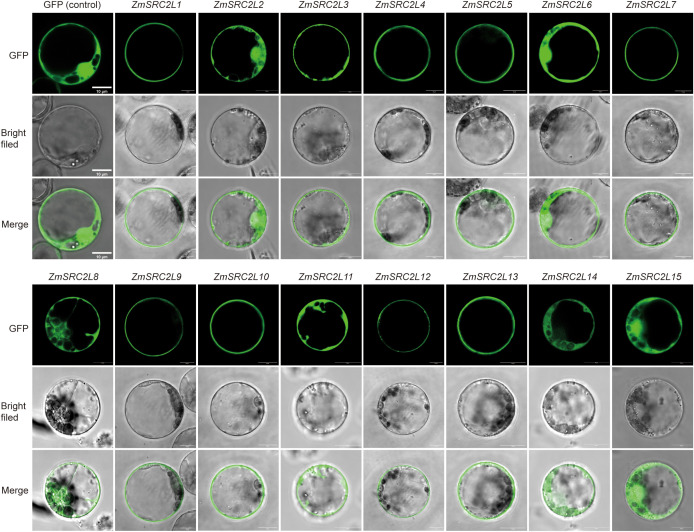
Subcellular localization of ZmSRC2L proteins.

Subcellular localization analysis revealed that most GFP-ZmSRC2L fusion proteins were predominantly distributed at the plasma membrane (**Figure 5**). This localization pattern is consistent with the biochemical characteristics of the C2 domain, which typically mediate Ca^2+^-dependent phospholipid binding and membrane association. Such features enable C2-domain proteins to function as molecular sensors and mediators of membrane-associated signal transduction. Therefore, plasma membrane-localized ZmSRC2Ls may play roles in Ca^2+^-dependent signaling or stress perception at the membrane interface, similar to other C2-domain proteins characterized in rice and Arabidopsis (Kim et al., 2003; Kawarazaki et al., 2013).

In addition, ZmSRC2L2, ZmSRC2L6, and ZmSRC2L15 exhibited dual localization at both the plasma membrane and the nucleus, suggesting that these proteins might mediate the crosstalk between membrane signaling and nuclear transcriptional regulation. Such dual localization could facilitate the integration of early membrane-derived Ca^2+^ signals with downstream transcriptional responses under abiotic stress conditions. Collectively, these results indicate that the diverse subcellular localization patterns of ZmSRC2L proteins reflect functional specialization, enabling maize to coordinate distinct signaling pathways in response to environmental stresses.

### 3.5 Expression patterns of *ZmSRC2L* genes in maize tissues and in response to abiotic stress

**Figure 6:**
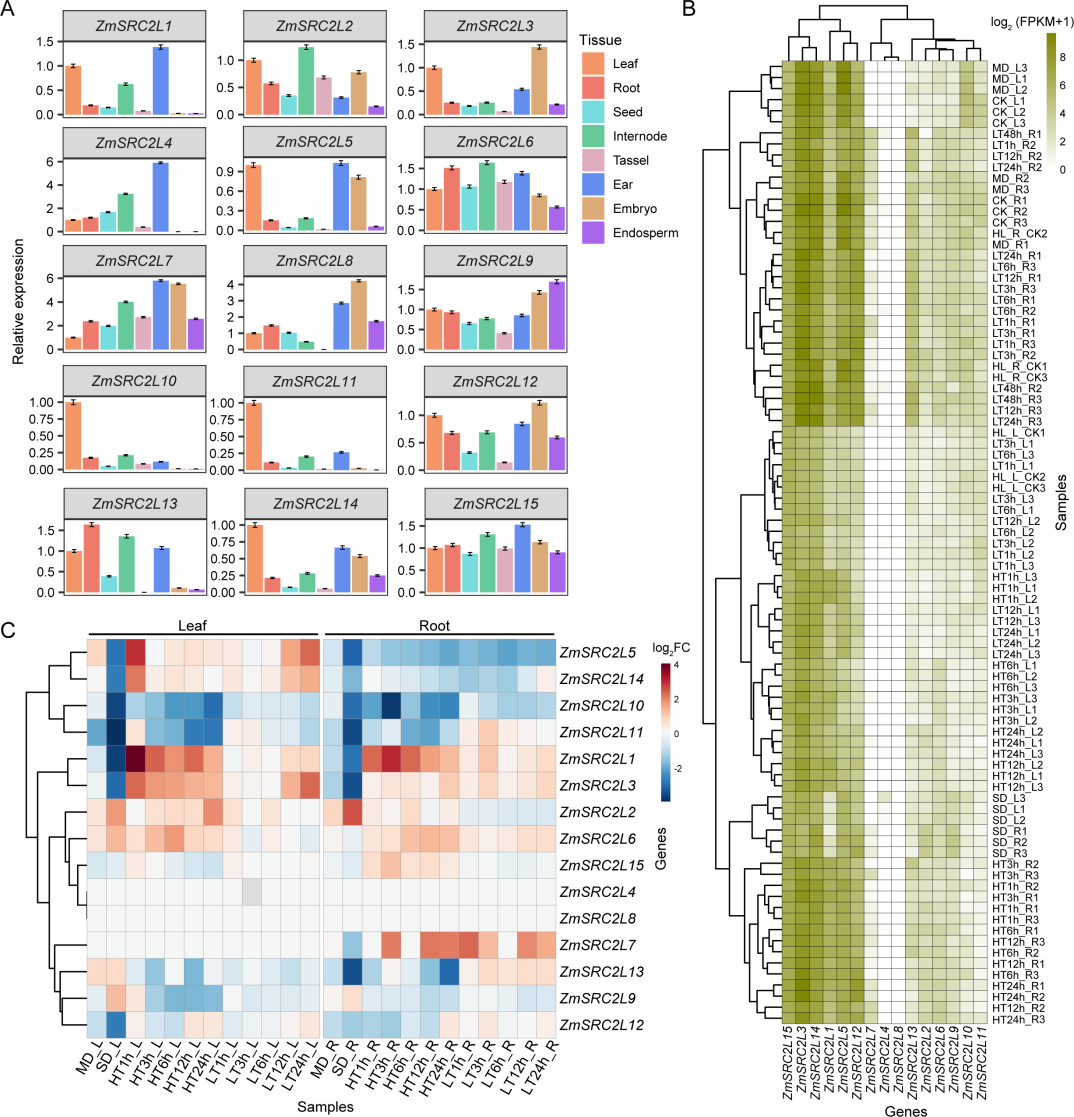
Expression patterns of *ZmSRC2L* genes in maize tissues and in response to abiotic stresses. **(A)** RT-qPCR–based tissue expression profiles of 15 *ZmSRC2L* genes across leaf, root, seed, internode, tassel, ear, embryo, and endosperm. Bars show relative expression for each gene, highlighting evident tissue specificity. Three biological replicates were performed. **(B)** Global expression heatmap of *ZmSRC2L* genes in leaves and roots under drought, heat, and cold treatments. Values are shown as log_2_(FPKM+1) with hierarchical clustering of both samples and genes. Condition abbreviations: CK, control; MD/SD, mild/severe drought; HT1h-HT24h, high temperature (heat) for 1–24 h; LT1h–LT24h, low temperature (cold) for 1–24 h; L/R, leaf/root. **(C)** Heatmap of differential expression dynamics (log_2_ fold change relative to CK) for *ZmSRC2L* genes across time courses of heat and cold and under mild versus severe drought. Red denotes up-regulation and blue denotes down-regulation; note the pronounced down-regulation of several members under severe drought and the time-dependent induction/repression under heat and cold.

To investigate the expression patterns of *ZmSRC2L* genes in maize, we performed RT-qPCR analysis on various tissues, including leaves, roots, internodes, tassels, ears, embryos, endosperms, and whole seeds. The results showed that *ZmSRC2L1*, *ZmSRC2L4*, *ZmSRC2L5*, *ZmSRC2L7*, and *ZmSRC2L15* were preferentially expressed in ears, *ZmSRC2L10*, *ZmSRC2L11*, and *ZmSRC2L14* were highly expressed in leaves, while *ZmSRC2L2* and *ZmSRC2L6* showed predominant expression in internodes and roots (**Figure 6A**). These distinct tissue-specific expression profiles suggest that the *ZmSRC2L* genes may play diverse and specialized roles in maize growth and development.

To elucidate the potential involvement of *ZmSRC2L* genes in abiotic stress responses, maize seedlings were subjected to drought (mild and severe), heat (1 h, 3 h, 6 h, 12 h, 24 h), and cold (1 h, 3 h, 6 h, 12 h, 24 h) treatments, followed by transcriptome sequencing of both leaf and root tissues. Samples under the same stress conditions clustered together, confirming the reliability of the transcriptomic data (**Figure 6B**). Group III genes in maize, including *ZmSRC2L1*, *ZmSRC2L3*, *ZmSRC2L12*, and *ZmSRC2L15*, exhibited relatively high expression (FPKM ≥ 40, an average across all tissues and stress conditions), whereas genes in Groups I, II, and IV showed lower expression (FPKM ≥ 4 and FPKM < 40). Notably, *ZmSRC2L7* and *ZmSRC2L8* (Group IV), as well as *ZmSRC2L4* (Group II) exhibited the lowest expression (FPMK < 4) among all members (**Figure 6B**). To further characterize the expression trends of ZmSRC2L genes under abiotic stresses, a heatmap of gene differential expression fold change was constructed across different time points (**Figure 6C**). The results revealed diverse expression patterns of *ZmSRC2L* genes under abiotic stress. Notably, *ZmSRC2L4*, *ZmSRC2L8*, and *ZmSRC2L15* did not show significant differential expression under these conditions. Under mild drought, the expression of these genes showed no significant changes, while under severe drought stress, all were significantly downregulated (**Figure 6C****;****Supplementary Table S6**). Under heat stress, *ZmSRC2L10* and *ZmSRC2L11* were markedly downregulated in both leaves and roots, while *ZmSRC2L1* and *ZmSRC2L3* were significantly upregulated in leaves. Interestingly, *ZmSRC2L5* and *ZmSRC2L14* were strongly induced in leaves during early heat exposure (1 h) and prolonged cold exposure (24 h). In contrast, heat and cold treatments repressed *ZmSRC2L5* expression in roots, whereas *ZmSRC2L14* showed no significant change (**Figure 6C****;****Supplementary Table S6**). Notably, *ZmSRC2L7* exhibits a low basal expression level, yet its expression in roots is markedly induced or suppressed under drought, heat, and cold stress. Collectively, the tissue- and stress-specific expression profiles of *ZmSRC2L* genes suggest that different members play distinct roles in maize growth, development, and environmental adaptation. These findings provide valuable insights into the potential functions of *SRC2L* genes in stress signaling and regulatory networks in maize.

### 3.6 Loss of function in *ZmSRC2L2* increases maize drought sensitivity

**Figure 7:**
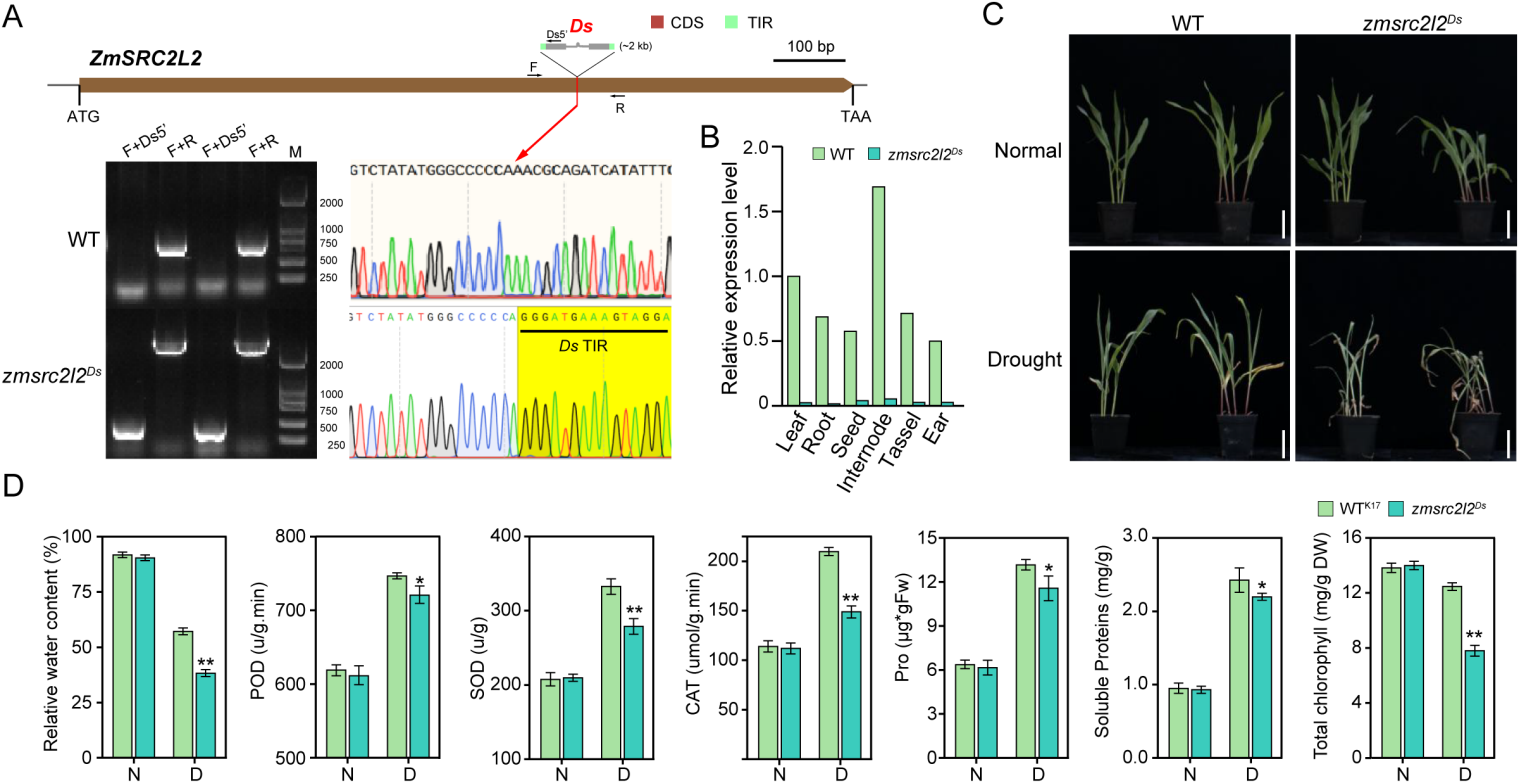
The loss function of *ZmSRC2L2* increased sensitivity to drought stress. **(A)** Identification of the insertion site of the Ds element within the *ZmSRC2L2* gene. **(B)** The expression levels of the *ZmSRC2L2* gene in *zmsrc2l2^Ds^* are significantly lower than those in WT across various tissues. **(C)** The plant phenotypes of the *zmsrc2l2^Ds^* and WT under drought stress conditions. **(D)** The activities of POD, SOD, and CAT, as well as the levels of RWC, Pro, soluble protein, and Chl were assessed in the *zmsrc2l2^Ds^* and WT under both normal and drought conditions. Three biological replicates were performed. *P< 0.05, **P < 0.01.

To investigate the role of the *ZmSRC2L* gene family in abiotic stress responses, we screened a previously established *Ac/Ds* transposon-tagged mutant library (Lyu et al., 2021) and identified a *Ds* insertion mutant of *ZmSRC2L2*. Sequence analysis revealed that a ~2-kb *Ds* element was inserted into the coding region, 692 bp downstream of the translation start site, resulting in a loss-of-function allele (**Figures 7A, B**). Phenotypic analyses at the seedling stage under drought, heat, and cold stress conditions revealed that the *zmsrc2l2^Ds^* displayed enhanced sensitivity specifically to drought stress (**Figure 7C**). Under drought conditions, the RWC of mutant leaves (38.25%) was significantly lower than that of the wild type (WT; 57.20%). Similarly, the *zmsrc2l2^Ds^* exhibited significantly reduced activities of POD, SOD, and CAT, as well as decreased accumulation of Pro and soluble proteins, compared to the WT under drought stress (**Figure 7D**). In contrast, no significant differences were observed between the mutant and WT in overall phenotype, RWC, antioxidant enzyme activities, or osmotic adjustment compounds under heat or cold stress (**Supplementary Figure S1**). These results demonstrate that *ZmSRC2L2* plays a critical role in conferring drought tolerance but not in mediating responses to temperature stress in maize, providing genetic evidence for functional diversification and stress-response specificity within the *ZmSRC2L* gene family.

## 4 Discussion

### 4.1 Identification and evolutionary characterization of *SRC2-Like* genes in maize

The *SRC2* gene family encodes C2 domain–containing proteins that function at the interface of Ca^2+^ signaling and membrane-associated processes, and accumulating evidence indicates that these proteins act as important regulators of plant stress responses (Nalefski and Falke, 1996; Wang, 2002). In dicotyledonous species, including *Arabidopsis thaliana*, soybean (*Glycine max*), pepper (*Capsicum annuum*), and *Nicotiana benthamiana*, SRC2 proteins have been implicated in regulation of reactive oxygen species (ROS) production, hormone-linked signaling pathways, and immune responses to pathogens (Kim et al., 2008; Kawarazaki et al., 2013; Liu et al., 2015; Chen et al., 2021). However, in contrast to their well-characterized functions in dicotyledonous plants, the roles of *SRC2-like* (*SRC2L*) genes in monocots, particularly in maize, remain poorly understood. This knowledge gap has limited our understanding of the mechanisms underlying maize responses to abiotic stress.

In this study, we identified 15 *SRC2L* genes in maize (*ZmSRC2L1*–*ZmSRC2L15*) (**Figure 1**) and, to our knowledge, conducted the first genome-wide analysis of this family in the Poaceae (**Figure 3**). Phylogenetic and collinearity analyses revealed that *SRC2L* genes are conserved across grasses but have undergone species-specific expansion, suggesting that gene duplication followed by subfunctionalization has contributed to their diversification. The strong purifying selection (Ka/Ks < 1) observed across orthologous pairs indicates evolutionary pressure to maintain essential C2-mediated signaling functions, consistent with reports on Arabidopsis CAR proteins (Rodriguez et al., 2014). Together, our results establish an evolutionary framework for the maize *SRC2L* gene family and provide a foundation for dissecting their roles in stress-adaptive signaling in cereals.

### 4.2 Promoter-mediated regulation of *ZmSRC2L* genes

Promoter cis-element analysis showed that *ZmSRC2L* promoters are enriched in light-, hormone-, and stress-responsive motifs (**Figure 4****;****Supplementary Table S4**). ABA-responsive ABREs were the most prevalent and widely distributed motifs, followed by MeJA-associated TGACG elements (**Figure 4**) (Yoshida et al., 2010; Zheng et al., 2017). Consistent with roles of ABRE and TCA modules in stress resilience (Li et al., 2020), we also identified multiple canonical stress-related cis-elements, including the stress response element (STRE), which is required for full heat-inducible activity of the *AtHsp90–1* promoter (Haralampidis et al., 2002), and the activation sequence-1 (as-1), a biotic stress–responsive element associated with oxidative signaling (Lam et al., 1989; Garretón et al., 2002). Together with the presence of MYB-binding sites, these motif features suggest that *ZmSRC2L* transcription may be regulated by multiple hormone- and ROS-related signaling pathways, thereby enabling these genes to play important roles under diverse environmental stress conditions.

Promoter cis-elements act in concert with transcription factors (TFs) to regulate the timing and magnitude of gene expression. In this study, we identified 16 TF families that are potentially involved in the regulation of *ZmSRC2L* genes (**Figure 4B**). Among these TF families, ERF factors were the most highly represented and were predicted to preferentially regulate the transcription of *ZmSRC2L15*. ERF TFs have been showed to modulate downstream transcriptional programs that regulate growth and development in Arabidopsis, as well as responses to diverse stress conditions (Liu et al., 1998; Jofuku et al., 2005; Castillejo and Pelaz, 2008). These observations support the hypothesis that ERF-mediated regulation of *ZmSRC2L* genes may contribute to developmental control and stress adaptation in maiz*e.* In addition, MYB TFs are key stress-responsive regulators that play important roles in controlling gene expression by modulating diverse biochemical pathways in plants under stress conditions (Wang et al., 2021; Zhang et al., 2025). Notably, *ZmSRC2L4*, *ZmSRC2L9*, *ZmSRC2L13*, and *ZmSRC2L15* were predicted to be regulated by multiple MYB factors (**Figure 4B**). Our findings suggest that *ZmSRC2L* genes may act as downstream components of ERF- and MYB-mediated transcriptional networks, integrating developmental and stress-related signals to fine-tune adaptive responses in maize.

### 4.3 Subcellular localization of ZmSRC2L proteins

The subcellular localization provides an important framework for inferring protein function. However, limited information on ZmSRC2L proteins has hindered mechanistic interpretation. Previous studies have shown that SRC2 proteins in other species display localization patterns consistent with roles in membrane-associated signaling. For example, pepper SRC2–1 localizes to the plasma membrane in protoplasts, and its complex with PcINF1 is likewise detected at the membrane (Kim et al., 2008; Liu et al., 2015). In contrast, the interaction between barley HvSRC2 and the Barley yellow dwarf virus 17K protein generates cytosolic signals (Wang et al., 2025), whereas Arabidopsis AtSRC2 is distributed between the cytosol and plasma membrane (Kawarazaki et al., 2013). Using transient expression assays in maize protoplasts, we found that most ZmSRC2L proteins predominantly localized to the plasma membrane, consistent with proposed roles for this family in membrane-associated signal transduction during environmental responses (Guo et al., 2024; Kawarazaki et al., 2013). Previous studies have reported that proteins consisting only an N-terminal C2 domain can localize to both the plasma membrane and nucleus, consistent with their Ca^2+^-binding capacity (Liu et al., 2018). Notably, ZmSRC2L2, ZmSRC2L6, and ZmSRC2L15 also exhibited multi-compartment localization, being observed at both the plasma membrane and in the nucleus (**Figure 5**). Differences in subcellular localization among ZmSRC2L proteins may be associated with sequence divergence in their non-conserved regions and represent an important factor contributing to functional diversification within this gene family.

### 4.4 Tissue-specific expression and stress responsiveness of *ZmSRC2L* genes

The expression patterns of *ZmSRC2L* genes across different tissues exhibited substantial diversity. Notably, *ZmSRC2L10* and *ZmSRC2L11* showed significantly higher expression in leaves than in other tissues, whereas *ZmSRC2L6*, *ZmSRC2L9*, and *ZmSRC2L15* displayed relatively uniform expression across tissues (**Figure 6A**). Our analysis revealed that tissue-specific expression differences among *ZmSRC2L* genes did not correspond to their phylogenetic classification groups, nor were they associated with the subcellular localization of the encoded proteins. Nevertheless, four genes belonging to group III (*ZmSRC2L1*, *ZmSRC2L3*, *ZmSRC2L12*, and *ZmSRC2L15*) exhibited high expression levels, whereas the majority of *ZmSRC2L* genes from groups I, II, and IV were classified as having moderate to low expression (**Figure 6B**). This finding suggests that the expression levels of *ZmSRC2L* genes under abiotic stress are associated with their phylogenetic classification, indicating that different *ZmSRC2L* groups may play distinct roles in abiotic stress responses. Previous studies have reported that SRC2 genes are involved in plant response to cold stress (Takahashi and Shimosaka, 1997; Kawarazaki et al., 2013). In our study, most *ZmSRC2L* genes exhibited either upregulation or downregulation in response to drought, heat, and cold stress. Furthermore, we observed that the expression patterns of individual *ZmSRC2L* gene under the same abiotic stress conditions occasionally differed between leaf and root tissues (**Figure 6C**). The *ZmSRC2L* gene family appears to play important roles in responses to multiple abiotic stresses and exhibits pronounced tissue-specific expression patterns, suggesting that its regulation of maize stress tolerance may involve complex and multilayered mechanisms. *ZmSRC2L15* exhibited consistently high expression across all examined maize tissues and under various stress conditions. Notably, differential expression analysis indicated that its expression was largely unaffected by external environmental changes, suggesting that the relatively stable expression of *ZmSRC2L15* may be required for maintaining normal growth and development in maize. These findings provide new insights into their biological functions and offer a foundation for their potential application in improving abiotic stress tolerance in maize breeding.

### 4.5 Drought-specific function of *ZmSRC2L2*

We found that *ZmSRC2L2* responds specifically to drought stress. Functional validation using a *Ds* insertion mutant demonstrated that loss of *ZmSRC2L2* markedly increased sensitivity to drought, whereas no significant phenotypic differences were observed under heat or cold stress (**Figure 7**; **Supplementary Figure S1**). The drought-specific role of *ZmSRC2L2* likely results from the unique combination of its promoter architecture, expression pattern, and conserved domain features. Promoter analysis revealed an enrichment of ABA- and drought-responsive cis-elements (ABRE and ARE) but few heat- or cold-responsive motifs, suggesting that its expression is predominantly controlled by ABA-dependent drought signaling pathways. Moreover, *ZmSRC2L2* exhibited the highest expression in internodes, a key tissue for water transport and structural stability, implying its involvement in maintaining hydraulic and physiological balance under water-deficit conditions. Although *ZmSRC2L2* shares the canonical C2 domain with other SRC2-like proteins, its dual localization at the plasma membrane and nucleus suggests a role as a Ca^2+^-mediated signal transducer, linking membrane-based drought perception with transcriptional regulation. Collectively, these features provide a mechanistic explanation for why *ZmSRC2L2* specifically confers drought tolerance but not heat or cold resistance in maize. Nevertheless, we acknowledge that the present data are primarily correlative and do not yet reveal the precise molecular mechanisms underlying *ZmSRC2L2*-mediated drought tolerance. In future studies, we will employ transgenic, gene-editing, and molecular analyses to validate its biological function and uncover the detailed signaling pathways involved in maize drought responses.

## Data Availability

The RNA-seq data have been deposited in the NCBI Sequence Read Archive (https://www.ncbi.nlm.nih.gov/sra, SRA) and can be accessed using the BioProject and SRA accession number PRJNA1393347.

## Supplementary material

Supplementary Figure 1 No significant differences were observed between the *zmsrc2l2^Ds^* and WT under heat or cold stress.

Supplementary Table 1 Sequences of primer and probe for molecular identification.

Supplementary Table 2 The summary of reads analysis from RNA-Seq data.

Supplementary Table 3 The *SRC2-like* genes in monocotyledon species.

Supplementary Table 4 Analysis of cis-acting elements within the promoter region of the *ZmSRC2L* genes.

Supplementary Table 5 Predicted potential regulatory transcription factors for *ZmSRC2L* genes.

Supplementary Table 6 The expression patterns of *ZmSRC2L* genes under abiotic stress conditions.
