# Supplementary figures and images for: Integrated genomic and transcriptomic analysis of maize SRC2-like genes highlights divergent roles in abiotic stress responses
Source: Front Plant Sci. 2026 Mar 2;17:1779434. doi: 10.3389/fpls.2026.1779434 (PMC12989361; doi:10.3389/fpls.2026.1779434)

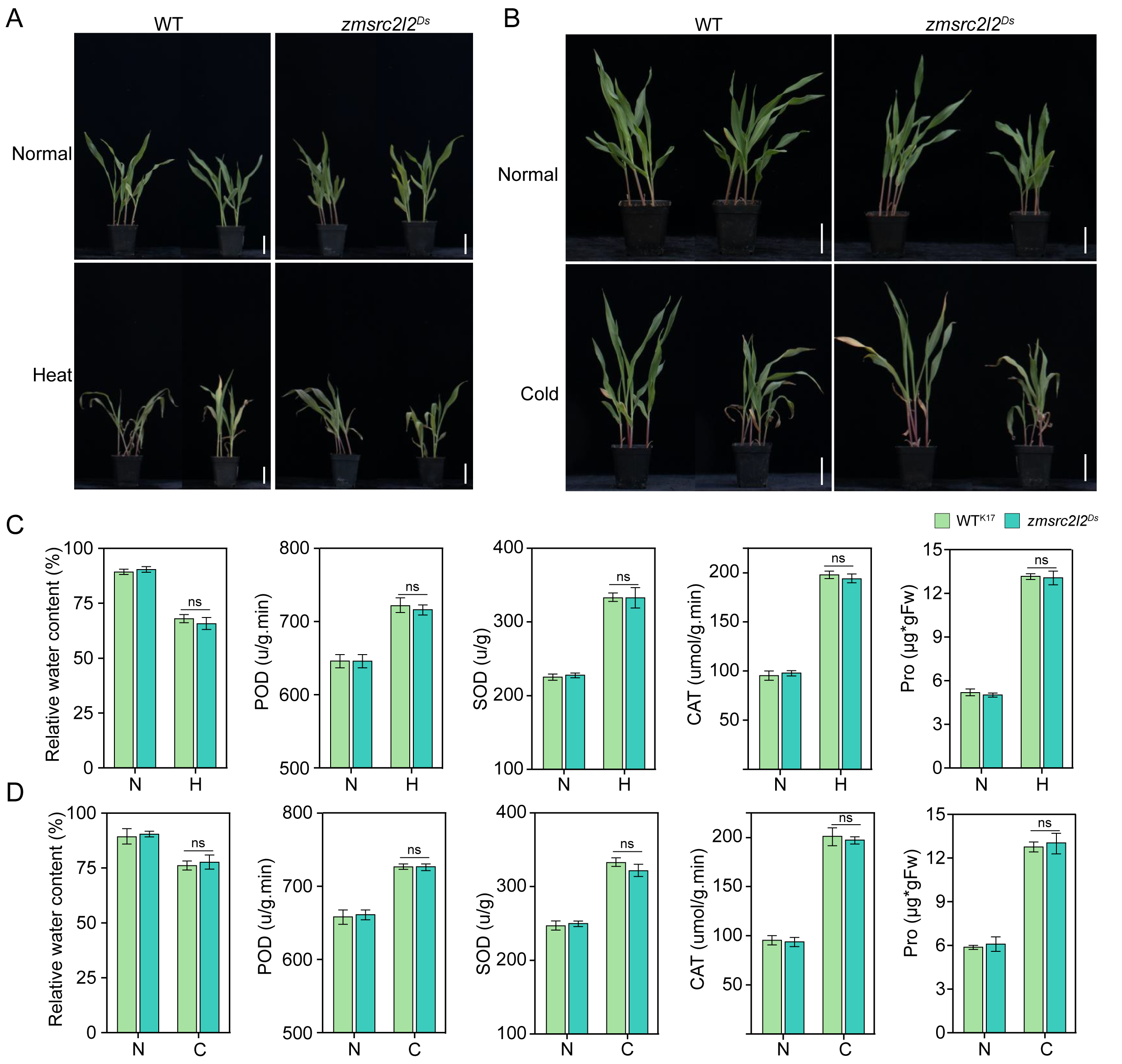

Supplement: Supplementary Figure 1 — No significant differences were observed between the zmsrc2l2Ds and WT under heat or cold stress. [file Image1.tif]
